# Cross‐Platform Demonstration of an Autonomous MRI Field Camera

**DOI:** 10.1002/mrm.70601

**Published:** 2026-10-02

**Authors:** Oskar Björkqvist, Klaas P. Pruessmann

**Affiliations:** ^1^ Institute for Biomedical Engineering ETH Zurich and University of Zurich Zurich Switzerland

**Keywords:** autonomy, field cameras, quality assurance, sequence detection, sequence development

## Abstract

**Purpose:**

Recent work has proposed self‐synchronization, self‐calibration, and means of sequence parsing to enhance the versatility of field cameras. The purpose of this paper is to expand this approach to more complex sequences and verify feasibility across distinct scanner platforms.

**Methods:**

A field camera was formed from six field probes in a rigid‐body arrangement and deployed in two commercial MRI scanners. Epoch folding for synchronization and the formation of sequence diagrams were expanded to nested sequences. Autonomous camera operation was used to record advanced sequences involving gradient‐echo trains, spin‐echo trains, and diffusion‐tensor encoding.

**Results:**

Autonomous camera operation was successful in both scanners, yielding sequence diagrams for all sequences explored.

**Conclusion:**

Autonomy of field cameras permits use across different MRI systems and degrees of sequence complexity. Potential applications range from method development and validation to sequence harmonization, multi‐center studies, and quality assurance.

## 1 Introduction

Field cameras for measuring dynamic magnetic fields in MRI scanners have seen increasing use over the past decade [1, 2]. Based on NMR, such cameras offer high sensitivity at the field strengths typically used in MRI scanners [3], capturing fine detail of intentional field dynamics as well as field imperfections and perturbations. Thus far, field cameras have been used mostly in research environments with MRI system expertise, relying on coordination with the MRI platform for calibration and synchronization.

Recently, it has been proposed to render field cameras more independent [4], using autonomous calibration and synchronization to eliminate the need for calibration sequences and triggers provided by the respective scanner. Self‐synchronization and sequence parsing have permitted the generation of full sequence diagrams despite the finite lifetime of probe signals. Thus far, these capabilities have been demonstrated for basic gradient‐echo and spin‐echo sequences, on one scanner platform. The objective of this technical note is to expand autonomous sequence recording to more complex sequences and verify platform independence by deploying an autonomous camera in different MRI systems, building mainly on the methods developed in previous work [4]. Greater sequence complexity is addressed by expanding epoch folding to nested sequences, including the wide range of methods using trains of gradient‐ or spin‐echos. For cross‐platform exploration, non‐steady‐state gradient‐echo [5], RARE [6], and diffusion tensor imaging sequences [7] are recorded on two different 3T scanners.

## 2 Methods

### 2.1 Measurement Setup

A description of measurement prerequisites and of the theoretical framework underlying position calibration and synchronization is provided in [4]. The overall workflow is summarized in Figure 1. Given a rigid‐body probe arrangement, the relative probe positions are determined by a one‐time measurement specific only to the probe module. Upon deployment in an MRI system, data acquisition is handled by a probe acquisition system (Skope [8]) and data processing is done using a custom Python framework that performs period finding, position calibration, and the generation of measurement triggers. Physical triggers are generated using a field programmable gate array (FPGA) that, in turn, is controlled by the Python software.

**FIGURE 1:**
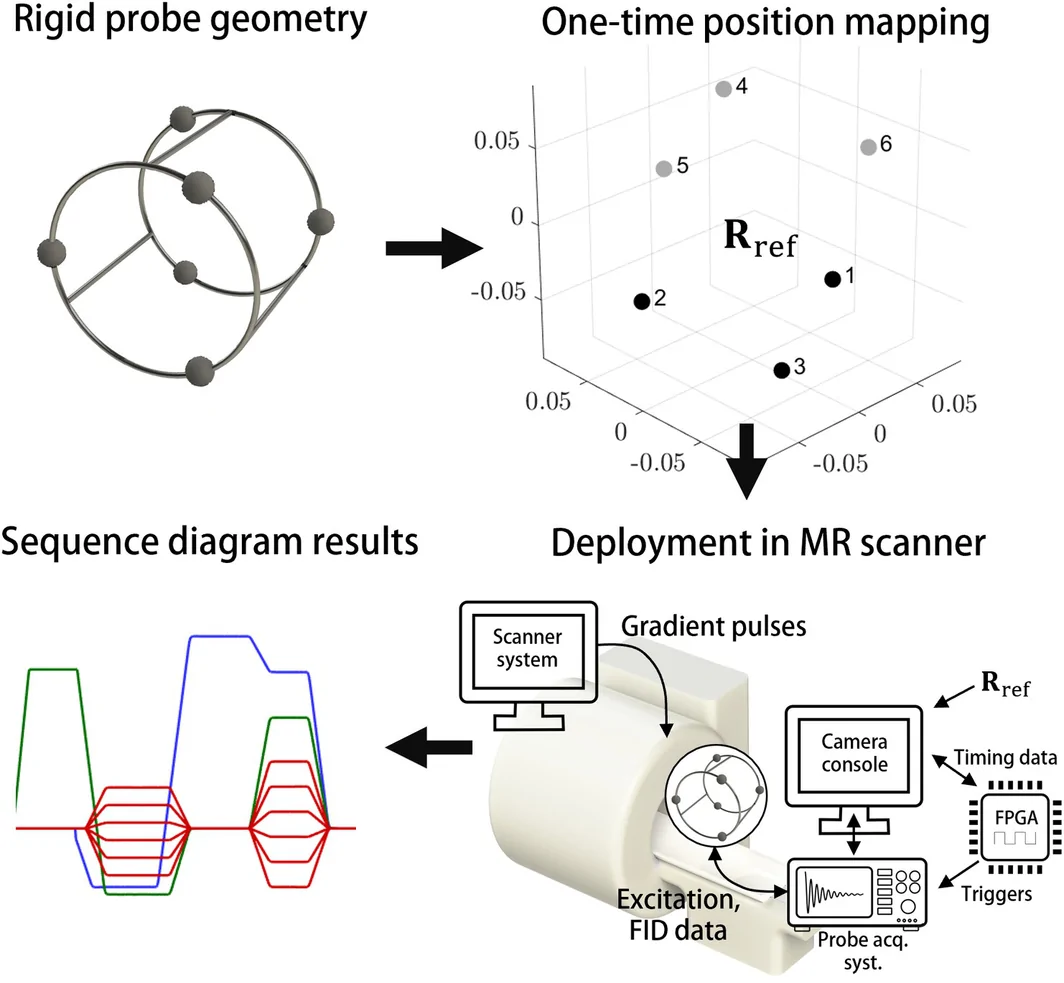
Workflow for autonomous sequence recording. Four or more field probes are arranged as a rigid body: In this case, six probes were mounted on the surface of a cylinder. Their relative positions are determined once and stored in a matrix $R_{\text{ref}}$. The probe module is placed in the MR scanner of interest, connected to a probe acquisition system, and operated by a console programmed to find sequence periodicity, perform position calibration, trigger field measurements, and compute sequence diagrams.

Most measurements were made using short‐$T_{1}$ probes ($T_{1}<$ 10 ms), allowing for rapid re‐excitation. The measurement duty cycle was kept within 15%–25% for all short‐$T_{1}$ probe recordings. While the method has been developed with such probes in mind, we demonstrate that it can also be applied using long‐$T_{1}$ (∼100 ms) probes. The short‐$T_{1}$ probes use 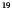
F‐NMR of hexafluorobenzene in sample capillaries of 1.3 mm inner diameter. The long‐$T_{1}$ probes are 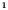
H‐based (H
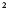
O), using 0.8 mm capillaries.

Measurements were carried out in two commercial whole‐body scanners: a Philips Elition X and a Siemens Cima.X. Field probes were tuned to the slightly different field strengths of these systems (3.0 T and 2.89 T, respectively). Each set comprised six probes distributed on the surface of a cylinder of 140 mm diameter. Where possible, RF power transmission by the scanner was disabled to allow for full observation of slice‐select gradients. Otherwise, data corrupted by RF pulsing was masked. Gradient field measurement during RF power transmission is equally possible [9, 10], but requires probe shielding and filtering not included in the setup used in this work.

### 2.2 Period Determination

To eliminate the need for trigger signals provided by the scanner, the probe acquisition system is made to operate independently by relying solely on the intrinsic periodicity of the running sequence for synchronization [4]. To identify the sequence period, a series of randomly timed field measurements is first performed, illustrated in Figure 2. Using these measurements, a cost function is calculated using epoch folding [11] to identify a fundamental sequence period. Epoch folding is a period‐finding method that folds time‐series data on itself at a candidate interval *T* to make periodic features stand out. This folding maps all observations onto the sequence period, forming time bins, and a cost function ∑|max−min| sums the per‐bin spreads across the bins. For demonstration purposes, the cost function was calculated using measurements from both short‐$T_{1}$ and conventional long‐$T_{1}$ probes to show that it is possible to do this independently of probe signal lifetime, Figure 2.

**FIGURE 2:**
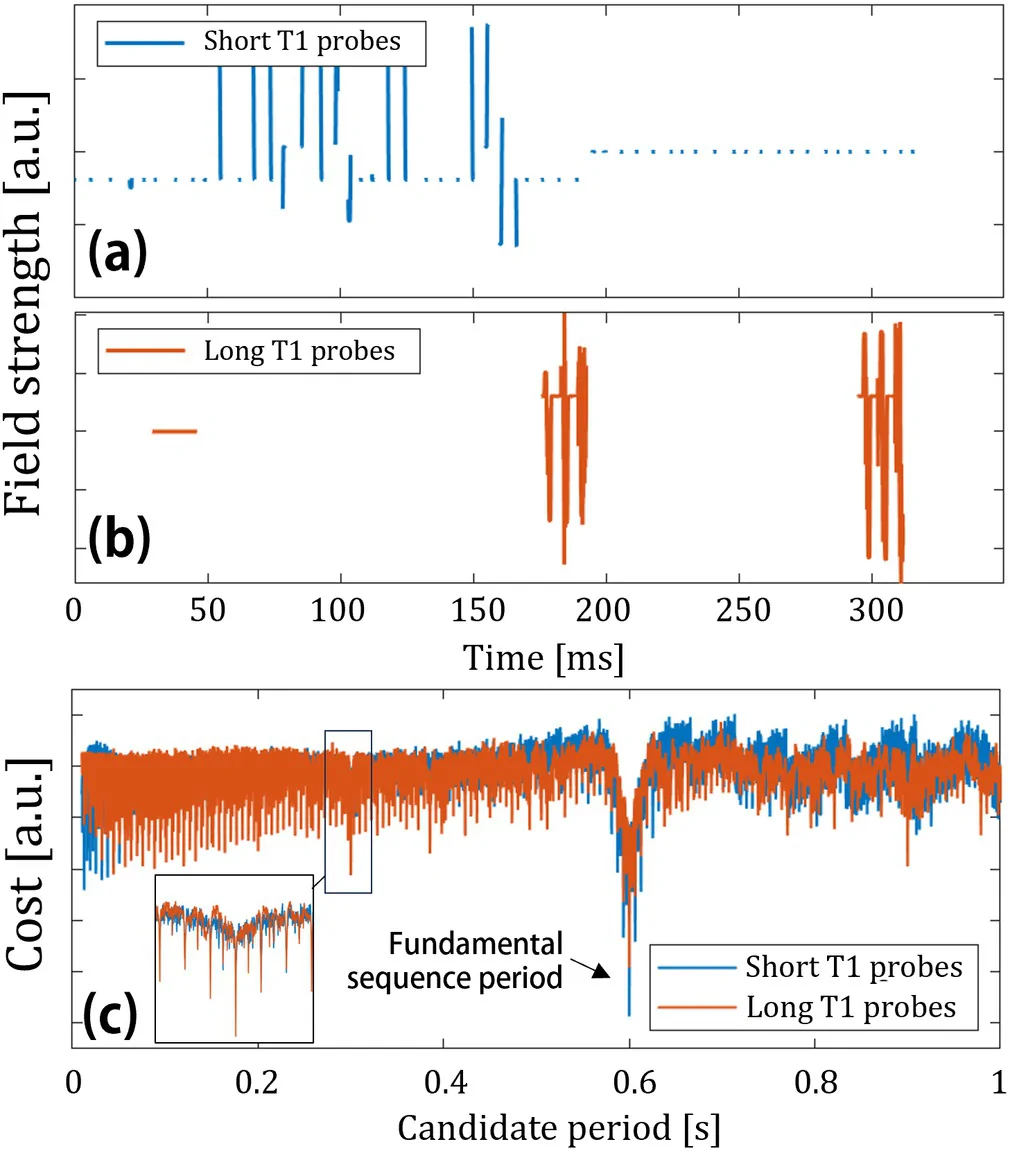
(a–b): Short‐ and long field snippet observations on a lab‐time grid of a Philips TFE sequence. (c) Epoch folding cost function. The sequence period at 600 ms visibly stands out. The comb like structure stems from the sequence's inner periodicity.

Previously demonstrated for basic sequences with simple periodicity [4], finding the fundamental sequence period is more involved for sequences with nested periodicities, where repeating substructures can obscure the fundamental period. Inner periodic structure tends to produce local minima in the epoch folding strategy. However, the fundamental period still dominates the cost function in the epoch folding approach. To illustrate this, recordings from a non‐steady‐state gradient‐echo sequence are displayed in Figure 2, together with the related epoch‐folding cost function. The comb structure present throughout is caused by the inner period. Despite this, the fundamental period is prominent enough to be distinguished from the sub‐periodic confounders.

Once the period has been established, the randomly timed snippets permit presenting the sequence in a pseudo‐continuous fashion [4]. On this basis, targeted measurements of arbitrary sequence features can then be carried out.

For nested sequences, these tools can be taken a step further. By selecting an inner loop, epoch folding can be performed a second time to reveal progressions such as phase encoding steps. Inner‐loop selection was done manually based on the pseudo‐continuous sequence diagram.

### 2.3 Probe Calibration

Probe calibration is performed by the method described in [4], applying its special form for a single rigid body of probes. A matrix **D** is constructed from the probe measurements, with each column representing a single probe. The measurements are assumed to follow a linear model

(1) D=GR+O

where we must know the per‐probe field offsets, forming constant columns of the matrix O, and the probe positions R to be able to determine the gradient waveforms, which form the columns of G. First, the offsets are accounted for by measurement during periods of gradient inactivity and subsequent subtraction from D, removing O from Equation (1). To solve for G and R, the probe positions are put into relation with a centered (i.e., row‐wise zero mean) reference measurement $R_{\text{ref}}$, through a rigid‐body transformation:

(2) $$R={\mathrm{QR}}_{\text{ref}}+{t1}^{⊤}$$

where the vector t represents global spatial coordinate offset and the vector 1 is filled with ones, one per probe. $R_{\text{ref}}$ only needs to be determined once for a given rigid‐body probe arrangement, by any suitable means. In the present work, this was done by MR measurement, using a custom sequence available on the Philips system. The orthogonal matrix Q is determined by exploiting orthogonality between slice‐select, phase‐encoding, and frequency‐encoding directions [4].

Substituting R in Equation (1) and right‐multiplying with $R_{\text{ref}}^{+}$ we obtain

(3) $$DR_{\text{ref}}^{+}=GQ\;R_{\text{ref}}R_{\text{ref}}^{+}+Gt1^{⊤}R_{\text{ref}}^{+}$$

where the term involving t reduces to zero,

(4) $${t1}^{⊤}R_{\text{ref}}^{+}=0$$

due to $R_{\text{ref}}^{+}$ being centered. The gradient matrix is thus independent of spatial probe offset and given by

(5) $$G=DR_{\text{ref}}^{+}Q^{-1}.$$

The final probe positions, including global offset, are then given by

(6) $$R=G^{+}D.$$

The procedure applies to any rigid‐body configuration of four or more probes that are not co‐planar. Once calibration is completed, the translation of probe data into gradient waveforms is done as conventionally with field cameras.

### 2.4 Sequence Measurements

To demonstrate the measurement capabilities, a representative set of sequences was selected and run on the Philips and Siemens systems. Wherever convenient, sequence parameters such as TR, TE, and flip angle were matched between the two scanners. Minor discrepancies may, however, remain due to scanner‐specific defaults, implementation differences, or console‐imposed constraints that were not adjustable.

Probe calibration was performed once in each scanner and not repeated between sequences. Synchronization is sequence‐specific and was carried out separately for each sequence. In each case, period detection was performed from randomized acquisitions, which simultaneously provided the data needed to produce pseudo‐continuous sequence diagrams. These data were not subject to further processing and measurement snippets were overlaid to form the complete sequence diagrams.

As a first example, non‐steady‐state 3D gradient‐echo sequences [5] were recorded (turbo field echo (TFE) on Philips, turbo FLASH (TFL) on Siemens). The sequence was configured to perform 3D phase encoding along the previously calibrated phase‐ and slice encoding directions. These sequences feature a nested periodic structure with echo trains embedded within a longer repetition period. Randomized snippets were acquired for an overview, supplemented, in the case of the short‐$T_{1}$ probes, by targeted acquisitions to observe variation of gradients across repetitions. The relevant sequence parameters are shown in Figure 3.

**FIGURE 3:**
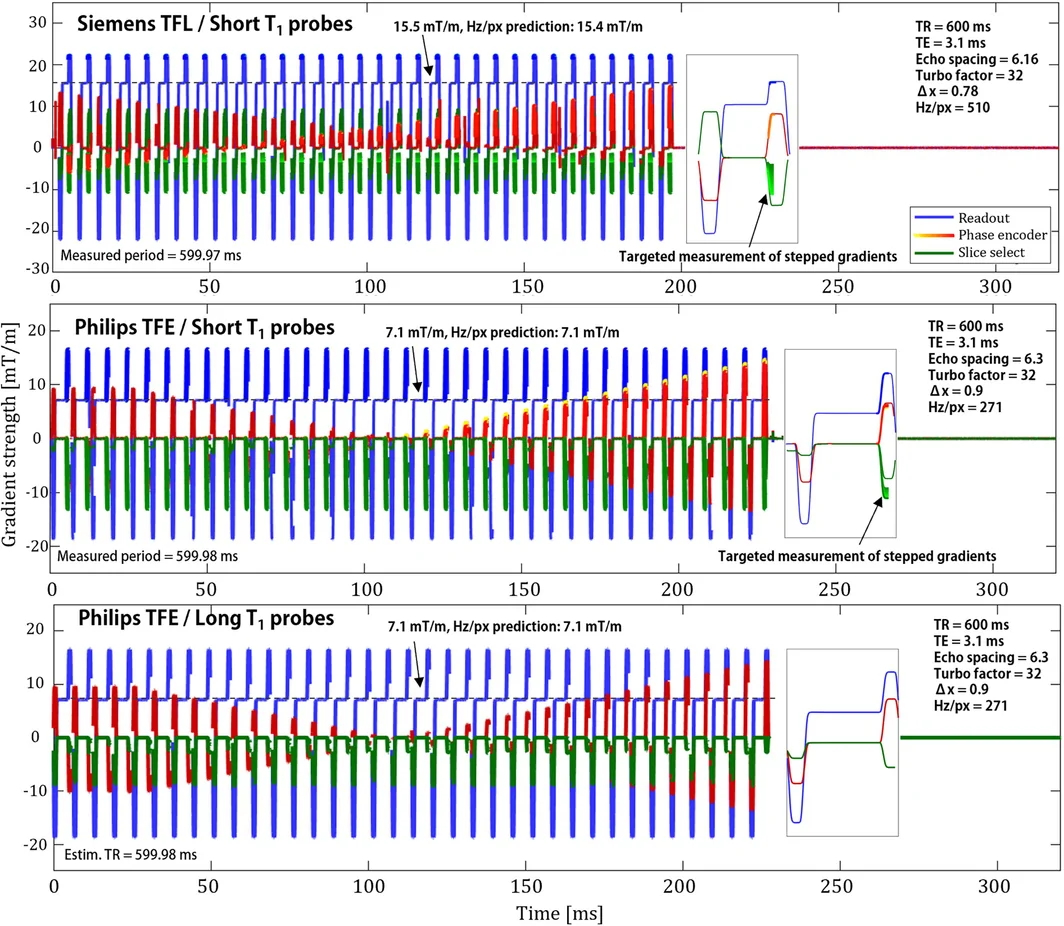
Top, middle: Siemens turbo flash (TFL) and Philips turbo field echo (TFE) pulse trains, respectively, observed using short $T_{1}$ probes. The insets feature a single instance of the inner loop and also include targeted measurements that highlight the evolution of the crusher gradient. Bottom: The same Philips TFE sequence observed using long $T_{1}$ probes. No targeted measurements were made due to the longer relaxation times of these probes.

As a second example, a 2D RARE [6] (TSE) sequence was recorded on both systems. On Siemens, snippets were randomized in time; to also demonstrate targeted measurements, on Philips, the period was determined in a first run and snippets were subsequently timed to guarantee full coverage of the period within four repetitions. We refer to this as *tiling* and it is done by orchestrating the measurements such that they deliberately cover a unique segment of the period, guaranteeing complete period coverage within 1/(measurement duty cycle) periods. An additional layer of epoch folding applied to the inner loop reveals the phase‐encoding ordering, enabling visualization of gradient evolution at the level of individual echoes, highlighting differences in phase‐encoding strategies.

Finally, DTI sequences [7] were run on both scanners. As with the previously described nested sequences, epoch folding was applied. Additionally, time‐gated separation was performed to account for varying direction of diffusion encoding. In this particular case, the strength of rapid probe re‐excitation becomes the most evident: Despite strong diffusion encoding gradients, short measurements (up to 1 ms in duration), re‐excited with a high duty cycle, allow for fully observing these full gradient trapezoids within a few repetitions. This enables full pseudo‐continuous display of the diffusion gradients for all directions.

With nominal gradient waveforms assumed unavailable, we employ a simple console‐based consistency check of the measurements. At the console, the readout gradient strength can be estimated as

(7) $$G_{\text{readout}}=\frac{f_{px}}{\gamma \Delta x},$$

where $f_{px}$ denotes the bandwidth per pixel and $\Delta x=\pi /k_{\max}$ the pixel size in the readout direction. Agreement indicates consistency between the scanner‐reported parameters and the measurements.

## 3 Results

The two measurements presented in Figure 2 are of the Philips TFE sequence, using short‐ and long‐$T_{1}$ probes, respectively. Epoch folding with a wide range of candidate periods yielded the shown graph of the folding cost function. The cost function exhibits a comb structure, reflecting the inner period of approximately 6 ms. The fundamental sequence period, however, is correctly identified as the global minimum at approximately 600 ms.

Sequence diagrams of the Philips TFE are shown in the middle and bottom rows of Figure 3, together with that of the Siemens TFL counterpart. According to nominal resolution and pixel bandwidth, the readout gradient strengths were 7.1 mT/m (TFE) and 15.4 mT/m (TFL), matching the measurements within less than 1%. In the Siemens case, the number of echoes equals the user‐defined turbo factor of 32, while the Philips pulse train is longer by four repetitions. The targeted measurements highlighted with color gradients in the two insets represent the progression of the crusher gradients for one echo within the pulse train. Stepping is seen to take place along the slice‐select axis and on a per‐shot basis, rewinding phase encoders in that direction. Stepping in the primary phase‐encode direction, in turn, is apparent in the sequence diagram per se and proceeds on a per‐echo basis.

In Figure 4, the two TSE recordings are shown. Similar overall structure along with certain differences in preparation and spoiling can be observed in (a) and (b). In (c–f), sub‐period epoch folding was performed on the parts of the sequence identified as inner loops. The color gradient follows two different structures: In (c) and (d), it follows lab time and is red earlier in the sequence, yellow later. In (e) and (f), each color is attributed to a position in the train. For validation, standard Skope calibration was performed and compared with the positions computed using our method. The mean relative error between the two methods was 0.37%, consistent with the related waveforms effectively coinciding in the inset in Figure 4. Note that this comparison could only be made on Philips because the Skope calibration sequence was not available on the Siemens system.

**FIGURE 4:**
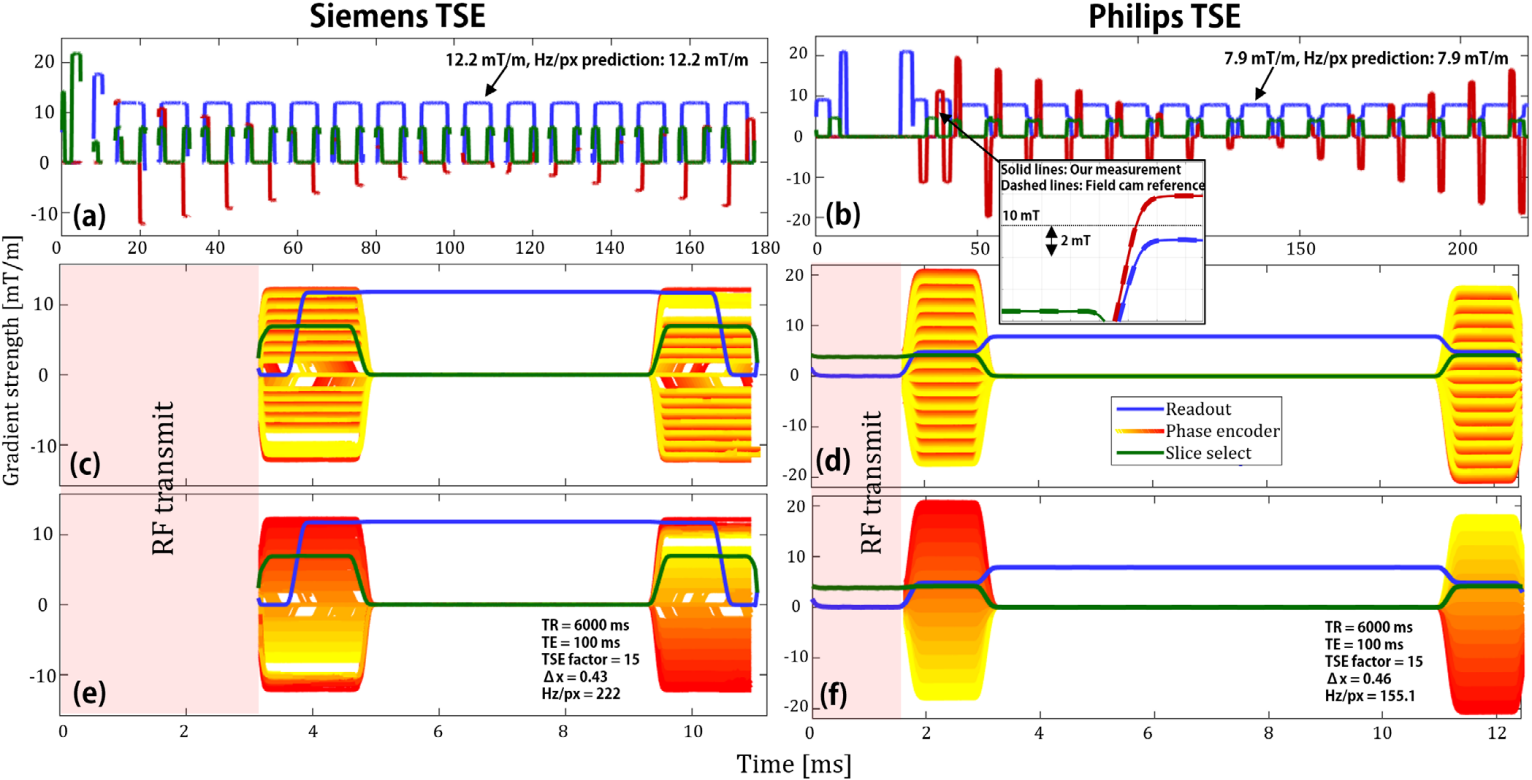
Overview of pseudo‐continuous sequence diagram results for turbo spin echo sequences on Philips and Siemens. (a) Siemens sequence diagram displaying the outermost period. (b) Philips sequence diagram displaying the outermost period. (c–d) Epoch folding of the inner periods with a color gradient matching a particular echo instance: Red is an earlier echo, yellow is a later one. (e–f) Epoch folding of the inner periods with a color gradient following lab time: Red is early in the sequence, yellow is later.

The two TSE sequences are seen to differ in terms of phase encoding. Variable duration is used for weak phase encoders in the Siemens sequence, while the Philips sequence exclusively varies the trapezoid magnitude. Gaps in the display of the variably timed gradients reflect the random sampling strategy, due to which some trapezoid start‐ and end points are missed. When of interest, full coverage could be achieved by targeted measurement.

We also want to point to the fact that the *TR* specified by the user (6000 ms in this case) does not necessarily match the repetition rate of the sequence: Individual slices can in principle be targeted at any multiple of the repetition rate to match a particular TR.

We again calculated an expected readout gradient strength using the nominal resolution and pixel bandwidth, highlighted at the top of the two figure columns. This metric again agreed well with the observations.

Lastly, the results from DTI sequence recordings are illustrated in Figure 5. After epoch folding, the individual diffusion‐encoding segments were separated according to their respective directions. The pseudo‐continuous representations including the diffusion encoding gradient trapezoids are visible (a) and (c). Capturing the large‐moment diffusion gradients is made possible by rapid re‐excitation of the probes and the repeating structure of the sequence. One immediate use of these readouts is to compute the corresponding *b* values, which yielded 797 and 798 $s/{\text{mm}}^{2}$ (nominal: 800 $s/{\text{mm}}^{2}$). In addition, the observed diffusion gradients reflect the directions of tensor encoding and the order in which they were executed, as shown in the sub‐figures (b) and (d). The two strategies for approximately uniform sampling of a sphere are evidently different: For Siemens, the encoding directions form the arrangement of hexagons and pentagons characteristic of a truncated icosahedron, creating the “football” pattern of diffusion tensor encoding [12, 13]. The Philips sequence achieves approximately uniform angular coverage in a different way, which is reminiscent of the electrostatic repulsion strategy for this purpose [14].

**FIGURE 5:**
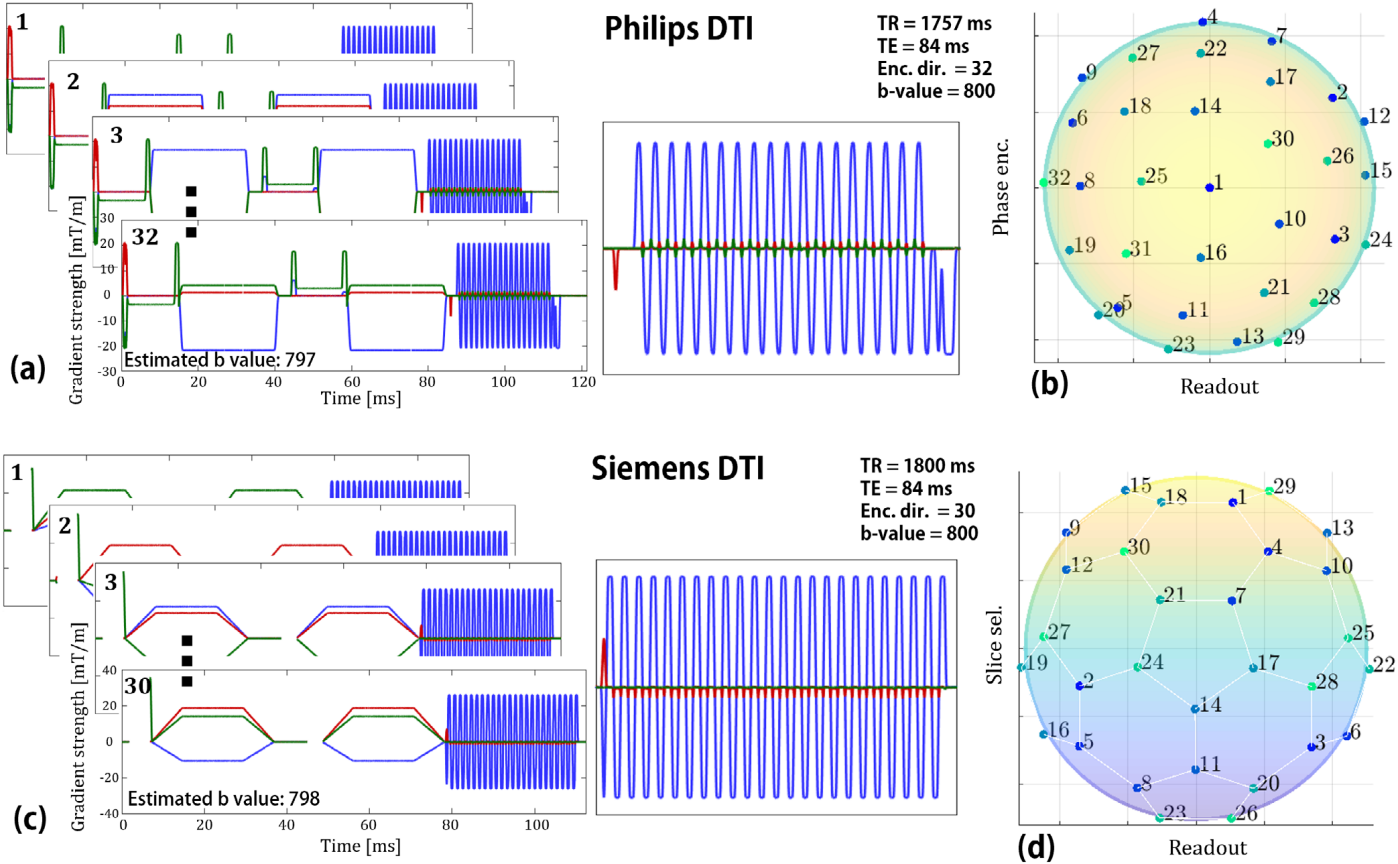
Recordings of diffusion tensor imaging (DTI) sequences. (a, c) Pseudo‐continuous sequence diagrams of Philips (top) and Siemens (bottom) DTI sequences implementing 32 and 30 encoding directions, respectively. The EPI readouts are magnified on the right hand side together with the requested sequence parameters. (b, d) Observed diffusion encoding directions plotted on the unit sphere. The apparent truncated‐icosahedron (“football”) sampling strategy in (d) is highlighted with white lines.

## 4 Discussion

These results confirm the ability to operate field cameras in a fully platform‐independent fashion. Removing the need for hardware and software interfaces simplifies camera use and greatly reduces the level of expertise and platform access required.

Complexity as encountered in advanced standard sequences has been found not to impede autonomy or the ability to obtain automated sequence schematics. The only prerequisite is sufficient presence of periodic elements, which the vast majority of common sequences exhibit. Non‐periodic gradients, for example, for phase encoding or diffusion weighting, are innate to imaging sequences but of limited relative power. Notable exceptions include purposely incoherent sequences, for example, for parametric mapping [15], and scans with physiological triggering [16]. In these cases, random tiling would still sample the sequence efficiently, but coherent sequence schematics will be more challenging to extract and present. A generic, but demanding, option for full, asynchronous coverage of any sequence is continuous field recording with alternating probe sets [10].

While automatic at the level of sequences to record, camera calibration does require prior knowledge of relative probe positions, which here was obtained via a custom sequence available on one of the scanners used. This is required only once for any given rigid‐body probe arrangement, for subsequent use in any scanner. Instead of MR, relative positions could also be obtained by other means such as, for example, X‐ray imaging or accurate mechanical construction. Alternatively to the six‐probe setup used in this work, any rigid‐body arrangement of at least four non‐coplanar probes can be employed. For more spatially constrained settings, it is an option to use sets of probe pairs (at least six) or triplets (at least three) [4], in which case relative positions up to rotation and reflection need to be known for each module individually. For probe sets with nine or more probes, it is additionally also possible to learn about higher order fields in the same way usually done with the conventional field camera procedure. This was however not considered in this work as the main goal was rather to produce sequence diagrams which only consider the first order field components.

Short‐$T_{1}$ probes have been favored in this work for rapid re‐excitation, while also demonstrating compatibility with longer $T_{1}$. The disadvantage of longer‐$T_{1}$ probes is lower effective duty cycle upon signal dephasing by gradients.

For the probes used in this work, interference by RF pulses from the scanner under study causes momentary saturation and signal drop‐out, which is why the plateaus of selection gradients are masked in some of the results. Otherwise, the gradient sequences were successfully recorded despite RF transmission. A logical next step will be to protect the probe chains by suitable shielding and filtering and incorporate a receive chain for concurrent RF pulse recording [9, 10, 17].

The work presented in this paper does not consider the method for the purpose of field camera augmented image reconstruction. This purpose comes with additional requirements that we do not address in this work. One such requirement is knowledge of the delay between gradient fields and raw data acquisition, which can be identified using customized sequences [18]. Automatic timing harmonization between a scanner and a field camera has also been achieved by mere computational means [19], suggesting that the present work in principle could be fused with reconstruction.

The methods applied in this work enable the continuous capturing of very strong gradients, a capability that is generally not offered by conventional field cameras. This is due to their conventionally longer NMR probe relaxation times (>100 ms) and the fact that epoch folding is not part of the conventional field camera toolkit. As such, we envision that some of the methods in this work could be used to study, for example, nonlinearities and eddy current effects on high‐performance gradient systems [20, 21, 22, 23, 24].

Vendor‐agnostic field measurement and sequence recording can be deployed in virtually any MRI scanner environment. While the demonstration is at the present limited to only two distinct scanners, the methods hinge only on a few basic assumptions about the scanner and sequences used: (1) Gradient spatial linearity and mutual orthogonality, (2) periodic imaging sequences, and (3) the availability of spin‐warp sequences. While we cannot say with certainty that the method is guaranteed to work on any scanner, these assumptions generalize very well across the vast majority of clinical scanners, which is why we would argue that demonstrating the method on other scanners may prove redundant.

A key implication is that sequences required to be identical for scientific or diagnostic purposes can be independently verified. While the method cannot be used to assess how data is processed past acquisition, it can be used to guarantee that sequences are identical at the physical level. This perspective aligns naturally with open, vendor‐neutral sequence development, where cross‐platform reproducibility is a central goal [25, 26, 27, 28, 29].

The ability to independently verify sequence diagrams and field fidelity holds promise also for quality assurance in longitudinal and multi‐site studies, where acquisition consistency should be maintained across scanner types, upgrades, and generations. This demand is arguably greatest for quantitative imaging, where measured parameters may be sensitive to sequence nuances.

In a broad sense, independent field cameras with cross‐platform capability may be a step toward more transparent, reproducible, and platform‐independent MR experimentation.

## Funding

This work was supported by the Swiss National Science Foundation (Bridge Discovery Grant 181023).

## Conflicts of Interest

The authors declare no conflicts of interest.

## Data Availability

Research data are not shared.

## References

[1] C. Barmet , N. De Zanche , and K. P. Pruessmann , “Spatiotemporal Magnetic Field Monitoring for MR,” Magnetic Resonance in Medicine: An Official Journal of the International Society for Magnetic Resonance in Medicine 60, no. 1 (2008): 187–197. 10.1002/mrm.21603 18581361

[2] B. E. Dietrich , D. O. Brunner , B. J. Wilm , et al., “A Field Camera for MR Sequence Monitoring and System Analysis,” Magnetic Resonance in Medicine 75, no. 4 (2016): 1831–1840. 10.1002/mrm.25770 25975352

[3] S. Gross , C. Barmet , B. E. Dietrich , D. O. Brunner , T. Schmid , and K. P. Pruessmann , “Dynamic Nuclear Magnetic Resonance Field Sensing With Part‐Per‐Trillion Resolution,” Nature Communications 7, no. 1 (2016): 13702. 10.1038/ncomms13702 PMC5146285 27910860

[4] O. Björkqvist and K. P. Pruessmann , “Autonomy for MRI Field Cameras: Synchronization, Self‐Calibration, and Sequence Detection,” Magnetic Resonance in Medicine 96, no. 1 (2026): 485–498. 10.1002/mrm.70339 PMC13156451 41926152

[5] A. Haase , “Snapshot FLASH MRI. Applications to T1, T2, and Chemical‐Shift Imaging,” Magnetic Resonance in Medicine 13, no. 1 (1990): 77–89. 10.1002/mrm.1910130109 2319937

[6] J. Hennig , A. Nauerth , and H. R. A. R. E. Friedburg , “RARE Imaging: A Fast Imaging Method for Clinical MR,” Magnetic Resonance in Medicine 3, no. 6 (1986): 823–833. 10.1002/mrm.1910030602 3821461

[7] P. J. Basser , J. Mattiello , and D. LeBihan , “MR Diffusion Tensor Spectroscopy and Imaging,” Biophysical Journal 66, no. 1 (1994): 259–267. 10.1016/S0006-3495(94)80775-1 PMC1275686 8130344

[8] Skope MRI , “Skope—Magnetic Resonance Technologies,” 2026 accessed March 2, 2026.

[9] D. O. Brunner , B. E. Dietrich , M. Çavuşoğlu , et al., “Concurrent Recording of RF Pulses and Gradient Fields–Comprehensive Field Monitoring for MRI,” NMR in Biomedicine 29, no. 9 (2016): 1162–1172. 10.1002/nbm.3359 26269210

[10] B. E. Dietrich , D. O. Brunner , B. J. Wilm , C. Barmet , and K. P. Pruessmann , “Continuous Magnetic Field Monitoring Using Rapid re‐Excitation of NMR Probe Sets,” IEEE Transactions on Medical Imaging 35, no. 6 (2016): 1452–1462. 10.1109/TMI.2016.2514608 26742126

[11] D. A. Leahy , W. Darbro , R. F. Elsner , et al., “On Searches for Pulsed Emission With Application to Four Globular Cluster X‐Ray Sources‐NGC 1851, 6441, 6624, and 6712,” Astrophysical Journal, Part 1 266 (1983): 160–170.

[12] M. Hasan Khader , D. L. Parker , and A. L. Alexander , “Comparison of Gradient Encoding Schemes for Diffusion‐Tensor MRI,” Journal of Magnetic Resonance Imaging: An Official Journal of the International Society for Magnetic Resonance in Medicine 13, no. 5 (2001): 769–780. 10.1002/jmri.1107 11329200

[13] P. G. Batchelor , D. Atkinson , D. L. G. Hill , F. Calamante , and A. Connelly , “Anisotropic Noise Propagation in Diffusion Tensor MRI Sampling Schemes,” Magnetic Resonance in Medicine 49, no. 6 (2003): 1143–1151. 10.1002/mrm.10491 12768593

[14] K. Jones Derek , A. Horsfield Mark , and S. Andrew , “Optimal Strategies for Measuring Diffusion in Anisotropic Systems by Magnetic Resonance Imaging. Magnetic Resonance in Medicine: An Official Journal of the International Society for,” Magnetic Resonance in Medicine 42, no. 3 (1999): 515–525. 10467296

[15] D. Ma , V. Gulani , N. Seiberlich , et al., “Magnetic Resonance Fingerprinting,” Nature 495, no. 7440 (2013): 187–192. 10.1038/nature11971 PMC3602925 23486058

[16] P. Lanzer , E. H. Botvinick , N. B. Schiller , et al., “Cardiac Imaging Using Gated Magnetic Resonance,” Radiology 150, no. 1 (1984): 121–127. 10.1148/radiology.150.1.6227934 6227934

[17] K. Kouame Ferdinand , A. Paisant , C. Aube , and H. Saint‐Jalmes , “Toward Accessible MRI: SDR4MR, a Simple RF Pulse Monitoring Technique Using an Inexpensive Software‐Defined Radio,” Magnetic Resonance Materials in Physics, Biology and Medicine 38, no. 5 (2025): 895–903. 10.1007/s10334-025-01249-z PMC12497666 40252109

[18] R. K. Robison , A. Devaraj , and J. G. Pipe , “Fast, Simple Gradient Delay Estimation for Spiral MRI,” Magnetic Resonance in Medicine: An Official Journal of the International Society for Magnetic Resonance in Medicine 63, no. 6 (2010): 1683–1690. 10.1002/mrm.22327 20512872

[19] P. I. Dubovan and C. A. Baron , “Model‐Based Determination of the Synchronization Delay Between MRI and Trajectory Data,” Magnetic Resonance in Medicine 89, no. 2 (2023): 721–728. 10.1002/mrm.29460 36161333

[20] T. K. F. Foo , E. T. Tan , M. E. Vermilyea , et al., “Highly Efficient Head‐Only Magnetic Field Insert Gradient Coil for Achieving Simultaneous High Gradient Amplitude and Slew Rate at 3.0 T (MAGNUS) for Brain Microstructure Imaging,” Magnetic Resonance in Medicine 83, no. 6 (2020): 2356–2369. 10.1002/mrm.28087 31763726

[21] G. Ramos‐Llordén , H.‐H. Lee , M. Davids , et al., “Ultra‐High Gradient Connectomics and Microstructure MRI Scanner for Imaging of Human Brain Circuits Across Scales,” Nature Biomedical Engineering 10, no. 2 (2026): 309–324. 10.1038/s41551-025-01457-x PMC12920100 40670720

[22] M. Weiger , J. Overweg , M. B. Rösler , et al., “A High‐Performance Gradient Insert for Rapid and Short‐T2 Imaging at Full Duty Cycle,” Magnetic Resonance in Medicine 79, no. 6 (2018): 3256–3266. 10.1002/mrm.26954 28983969

[23] M. Weiger , J. Overweg , F. Hennel , et al., “A Unipolar Head Gradient for High‐Field MRI Without Encoding Ambiguity,” Magnetic Resonance in Medicine 95, no. 2 (2026): 1266–1278. 10.1002/mrm.70098 PMC12681299 41024510

[24] D. A. Feinberg , A. J. S. Beckett , A. T. Vu , et al., “Next‐Generation MRI Scanner Designed for Ultra‐High‐Resolution Human Brain Imaging at 7 Tesla,” Nature Methods 20, no. 12 (2023): 2048–2057. 10.1038/s41592-023-02068-7 PMC10703687 38012321

[25] K. J. Layton , S. Kroboth , F. Jia , et al., “Pulseq: A Rapid and Hardware‐Independent Pulse Sequence Prototyping Framework,” Magnetic Resonance in Medicine 77, no. 4 (2017): 1544–1552. 10.1002/mrm.26235 27271292

[26] C. Cordes , S. Konstandin , D. Porter , and M. Günther , “Portable and Platform‐Independent MR Pulse Sequence Programs,” Magnetic Resonance in Medicine 83, no. 4 (2020): 1277–1290. 10.1002/mrm.28020 31631406

[27] H. J. M. M. Mutsaerts , M. J. Van Osch , F. O. Zelaya , et al., “Multi‐Vendor Reliability of Arterial Spin Labeling Perfusion MRI Using a Near‐Identical Sequence: Implications for Multi‐Center Studies,” NeuroImage 113 (2015): 143–152. 10.1016/j.neuroimage.2015.03.043 25818685

[28] H. Mirzaalian , A. Pierrefeu , P. Savadjiev , et al., “Harmonizing Diffusion MRI Data Across Multiple Sites and Scanners,” in International Conference on Medical Image Computing and Computer‐Assisted Intervention (Springer, 2015), 12–19. 10.1007/978-3-319-24553-9_2 PMC5045042 27754499

[29] A. Karakuzu , L. Biswas , J. Cohen‐Adad , and N. Stikov , “Vendor‐Neutral Sequences and Fully Transparent Workflows Improve Inter‐Vendor Reproducibility of Quantitative MRI,” Magnetic Resonance in Medicine 88, no. 3 (2022): 1212–1228. 10.1002/mrm.29292 35657066

